# Novel Implications of Nanoparticle-Enhanced Radiotherapy and Brachytherapy: Z-Effect and Tumor Hypoxia

**DOI:** 10.3390/metabo12100943

**Published:** 2022-10-05

**Authors:** Runze Zhou, Di Zhao, Narasimha M. Beeraka, Xiaoyan Wang, Pengwei Lu, Ruixia Song, Kuo Chen, Junqi Liu

**Affiliations:** 1 Department of Radiation Oncology, The First Affiliated Hospital of Zhengzhou University, Zhengzhou 450000, China; 2 Endocrinology Department, The First Affiliated Hospital of Zhengzhou University, Zhengzhou 450000, China; 3 Department of Pharmaceutical Chemistry, Jagadguru Sri Shivarathreeswara Academy of Higher Education and Research (JSS AHER), Jagadguru Sri Shivarathreeswara College of Pharmacy, Mysuru 570015, India; 4 Department of Human Anatomy, I.M. Sechenov First Moscow State Medical University of the Ministry of Health of the Russian Federation (Sechenov University), 119991 Moscow, Russia; 5 Department of Breast Surgery, The First Affiliated Hospital of Zhengzhou University, Zhengzhou 450000, China

**Keywords:** tumor cell metabolism, radiotherapy, brachytherapy, Z-effect, nanoparticles, tumor hypoxia, clinical translation

## Abstract

Radiotherapy and internal radioisotope therapy (brachytherapy) induce tumor cell death through different molecular signaling pathways. However, these therapies in cancer patients are constrained by dose-related adverse effects and local discomfort due to the prolonged exposure to the surrounding tissues. Technological advancements in nanotechnology have resulted in synthesis of high atomic elements such as nanomaterials, which can be used as radiosensitizers due to their photoelectric characteristics. The aim of this review is to elucidate the effects of novel nanomaterials in the field of radiation oncology to ameliorate dose-related toxicity through the application of ideal nanoparticle-based radiosensitizers such as Au (gold), Bi (bismuth), and Lu (Lutetium-177) for enhancing cytotoxic effects of radiotherapy via the high-Z effect. In addition, we discuss the role of nanoparticle-enhanced radiotherapy in alleviating tumor hypoxia through the nanodelivery of genes/drugs and other functional anticancer molecules. The implications of engineered nanoparticles in preclinical and clinical studies still need to be studied in order to explore potential mechanisms for radiosensitization by minimizing tumor hypoxia, operational/logistic complications and by overcoming tumor heterogeneity in radiotherapy/brachytherapy.

## 1. Introduction

Radiotherapy (RT) is one of the most effective anticancer strategies, and is widely used in the standardized treatment protocols against multiple solid tumors. Radiotherapy is useful in targeting benign and malignant tumors in the central nervous system and endocrine system [1]. According to the latest research, more than 470,000 patients have been receiving radiotherapy each year due to cancers in the United States [2]. During radiotherapy, ionizing radiation beams, high-energy X-rays, γ-rays, or electron beams are irradiated onto the tumor tissues to target cancer cells [3]. Radioresistance is induced due to recurrence or clinical progression [4]. Some tumor types or lesions may have low sensitivity to the tumoricidal effects of radiation through multiple mechanisms including hypoxia and accelerated repopulation of tumor cells during radiotherapy; this further leads to accumulation of the resistant tumors capable of surviving against radiotherapy. Therefore, targeting radioresistant tumors has become a significant research topic in the field of radiation oncology [5,6,7].

Many cancer patients receive RT as a primary mode of treatment or adjuvant therapeutic modality in addition to chemotherapy. Higher dosages of radiation could ablate cancer cells, subsequently mitigating the disease recurrence of cancer progression [8,9,10]. Systemic RT, external beam RT, and internal RT are the three kinds of therapies used during treatment. In the systemic radiation therapeutic modality, targeted RT can deliver radiolabeled isotopes with carrier molecules. These components can effectively bind to the receptor moieties overexpressed across cancer cells. Internal radiation treatment, also referred to as brachytherapy (BT), is a prominent method of placing radioactive sources in close proximity to the tumor volume. This method can deliver a higher dose of radiation precisely across the tumors while hindering radiation effects on the surrounding normal tissues. The radioactive source inserted into the body during brachytherapy generates the X-rays into small, well-defined lesions, which thereby spare more normal tissue from exposure [11]. The exposure of tumor tissues to high-energy photons in external beam RT is performed across the tumor volume through the placement of radiation-generating moieties outside the patient’s body [12]. BT is very cost-effective in treating solid tumors due to limited side effects [13].

Technological advances in the field of nanomedicine have led to the synthesis of nanoparticle-based therapies or adjuvants in recent decades. Nanocarriers have been introduced into the field of cancer therapy for effective drug delivery due to their nanoscale size, high surface-to-volume ratio, and favorable physical/chemical characteristics [14,15,16]. The nanoparticles used in cancer therapy include several materials such as organic, inorganic, lipid, protein, and glycan compounds, as well as various synthetic polymers. Copious recent research studies have reported that free radical generation (ROS) is a vital process involved in killing tumor cells during radiotherapy; the introduction of nanoparticles along with radiotherapy to promote free radical generation has become a great opportunity for the combination of nanotechnology and radiation oncology [17,18,19]. Many radiosensitizing high-Z metal nanoparticles exhibit a significant ability to enhance ionization-induced ROS generation and are beneficial for avoiding the side effects of radiotherapy [8]. Nanoparticles have long been widely used in multifarious therapeutic and PET as contrast mediums for enhanced imaging, and as radiation dose enhancers and as radiation sensitizers. In addition to the classical role of enhancing dose enrichment in tumor lesion regions, recent advances in this field have increasingly focused on modulating the tumor cell microenvironment to achieve effective treatment [20,21].

The immune responses during radiotherapy could induce the killing of tumor cells to a great extent; as a result, nanoparticle-enhanced cancer immunotherapy has been gaining substantial attention in recent years [22,23,24]. Furthermore, tumor-associated antigens are released after radiotherapy-induced cancer cell death to trigger a series of immune responses for inflammatory microenvironment remodeling [25]. Radiotherapy induces the maturation of dendritic cells and other immune responses in tumor-bearing mice injected with nanoparticles. In addition, a combinatorial regimen of RT with nanoparticles could impair the activity of Tregs by blocking the CTLA4 checkpoint for immunoadjuvant activity [26]. The abscopal effect caused by local RT also becomes a systematic anti-tumor immune response of RT in combination with immune checkpoint blockade [27]. Thus, several immunological implications of RT in combination with nanoparticles against tumor cells have been described in the literature.

Radiotherapy can mitigate cancer cell division through the induction of necrosis, autophagy or senescence [28,29,30]. During apoptosis and necrosis, the damaged cells undergo death and senescence. ROS generation fosters oxidative stress accompanied by necrosis and substantially releases cellular content, resulting in a higher inflammatory response. For instance, membrane-targeted nanoparticles can induce ROS-mediated necrosis through cell membrane destruction [31]. Massive ROS generation is linked to cell death or apoptosis mediated by mitochondria cell death receptors/endoplasmic reticulum stress [32]. Autophagy is another process in which cells undergo self-destruction depending on the surrounding conditions [32]. However, the tumor cells can acquire resistance through transient responses such as DNA repair and antioxidant signaling against low intensity of radiation-induced ROS generation. As water is the significant constituent of cells inside the human body, the radiation effect of X-rays induces radiolysis of water molecules and generates radiolytic products, which further enhances the ROS-induced oxidative stress across tumor volume [33,34]. During these sequential events, the hypoxic environment during radiotherapy where the unbound oxygen is scarce is a significant mechanism for radioresistance and ineffective radiotherapy [35]. Thus, the radiation-induced ROS production is significantly limited by the hypoxic environment across the tumors due to inadequate blood circulation. However, the administration of nanoparticle-based therapy along with radiotherapy can enhance the radiosensitization during tumor hypoxia, but this requires substantial research studies to explore the cellular mechanisms underlying tumor hypoxia.

Nanoscale-range radiopharmaceuticals can undergo efficient local diffusion from their injection site and could be conducive to the equal dose distribution across tumor volume. Notably, high Z-nanoparticles can be applied along with radiotherapy or brachytherapy as multifunctional carriers for effective delivery of radioisotopes. Thus, this kind of high-Z nanoparticle can foster self-sensitization and limit the overall radioactive dose when compared to conventional brachytherapy. However, reports related to the efficacy of combinatorial implications for modulating the Z-effect and tumor hypoxia are minimal. Furthermore, rapid advances in synthesizing smaller agents such as nanoparticles could induce a higher therapeutic index. For instance, nanoscale brachytherapy may enhance the therapeutic outcomes with smaller agents such as ^177^Lu-labeledgold NPs [36]. Another study described the formulation of peptide-based NPs with ‘Arg-Gly-Asp (RGD)’ with ‘radiolabeled gold NPs’ (^177^Lu-AuNP-RGD), which induced effective anti-tumor effects in in vivo models of alpha(v)beta(3)-integrin-positive C6 gliomas [37]. Furthermore, the radiolabeled ^177^Lu-T-AuNP impregnated with panitumumab can impair the EGFR(+) triple negative breast cancers [38]. Tumor-suppressive effects are typically higher with radiolabeled ^177^Lu-DTPA-pAuNS in combination with photothermal therapy than the individual therapy of ^177^Lu-DTPA-pAuNS in head and neck squamous cell carcinoma [39]. Another study described the efficacy of ‘PAMAM-G4–(^177^Lu–dendrimer)–bombesin–folate’ for targeting gastrin-releasing peptide receptors as well as folate receptors in lung cancer cells [40]. Hence, ^177^Lu-labeled nanosystems could be considered as efficient theranostic radioactive components. Tumor cells express prostate-specific membrane antigen (PSMA) and fibroblast activation protein (FAP) is expressed across the tumor microenvironment. The radiolabeled ^177^Lu_2_O_3_-iPSMA and ^177^Lu_2_O_3_-iFAP can induce the impairment of cancer progression in colorectal liver metastases [41]. Another report described the efficacy of ^177^Lu_2_O_3_-iPSMA NPs along with radiotherapy to target PSMA-expressing cancer cells of hepatocellular carcinoma [42]. In this review, we aim to focus on the current development of nanoparticle technology combined with clinical radiation oncology for promoting radiosensitization and limiting radioresistance and tumor hypoxia. In addition, we also address the opportunities and challenges for clinical translation of new generation nanoparticle technologies in radiation therapy.

### Literature Search

We searched several databases, such as Pubmed, Medline, eMedicine, National Library of Medicine (NLM), and ReleMed, for published reports and articles, including reviews and original articles. Subsequently, we gathered information pertinent to the updated novel implications of nanotechnology along with radiotherapy or brachytherapy for mitigating radioresistance or tumor hypoxia, and their implications in clinical translation.

## 2. The History, Current Status, and Limitations of Radiation Oncology

Wilhelm Röntgen in 1895 discovered X-ray radiation, which could be applied in the fields of diagnostic and therapeutic strategies. However, the determination of optimal X-ray doses has always been a significant topic in radiation oncology [43]. In order to achieve precise radiation dose control to avoid radiation-induced side effects, high-energy X-ray sources, linear accelerators, fractionated dose applications and collimation technologies were introduced into clinical radiotherapy. Although the development of these techniques has alleviated the side effects to certain extent, there are limitations associated with dose distribution management and the accuracy of radiotherapy planning, because in addition to tumor tissues, current dosage regimens induce damage to normal tissues. Therefore, researchers have been exploring ways to enhance the efficacy of radiotherapy and reduce irradiation-induced damage in normal tissue [44,45,46]. In the year 1920, researchers in Germany reported the radiation-induced alteration of protein levels during normoxia conditions. A strong correlation was observed between radiosensitivity and oxygen level, which was proven by Thomlinson and Gray in 1955 [47]. Several scientific studies describe hypoxia in tumors as strongly associated with radioresistance because oxygen is indispensable in the free-radical damage induced to target DNA. Free radicals are generated inside the tumor cells through absorption of radioactive rays, which results in cell death via apoptosis and/or necrosis. The cytotoxic effect of radiation can be amplified by two or three times under oxygen-enriched conditions [48]. Due to the lack of targeted drugs to improve the anoxic environment around tumor cells, researchers have been focusing on emerging nanomaterial technologies for targeting tumor hypoxia. The application of conventional radiotherapy may not effectively inhibit the growth of distantly metastasizing tumor cells and control the systemic progression of tumors, which is often considered as the ultimate cause of cancer-related mortality [49]. Therefore, it is crucial in clinical radiation oncology to improve the effect of radiotherapy using theranostic radioactive nanosystems in hypoxic microenvironments and deliver systemic anti-tumor effects.

## 3. The Innate Properties of Nanomaterials

In recent years, the existing technical barriers in the clinical radiation oncology sector have required implementation of novel technologies. Multi-mode radiotherapy combined with novel technology is gradually enhancing the efficacy of comprehensive treatment and management of malignant tumors. These therapies include concurrent chemotherapy and radiotherapy, perioperative radiotherapy, and radioactive particle implantation. The significant attributes of nanomaterials determine their unique advantages as an adjunct in radiotherapy to target tumor cells. Poor water solubility enormously limits bioavailability and therefore hampers the development of novel anti-tumor agents [50]. The lipid-based nanocarrier systems can be used in the delivery of anti-tumor drugs with insoluble properties [51,52]. This barrier can be tackled by enveloping the anti-tumor agents in a hydrophilic nanocarrier, which can increase the chemical stability [53]. For instance, wortmannin is a PI3K inhibitor and radiosensitizer, but its usage is limited in clinical applications due to its poor solubility and chemical stability; however, its solubility can be increased from 4 mg/L to 20 g/L, while increasing its stability in vivo, when it is formulated with nanoparticles [54]. The engineered nanoparticles with radiosensitizers could enhance the biocompatibility of anti-tumor drugs by improving the water solubility and chemical stability across tumor volume. Tumor-homing nanoparticles containing high-Z elements (gold, gadolinium oxide) are attributed to promoting radiation energy around tumors and thereby enhancing the cytotoxic effects of radiotherapy [55,56,57]. The radiotherapy engineered with nanomaterials enhances the ROS-mediated oxidative stress across tumor regions, where the mitigation of oxidative damage to the surrounding healthy tissue has a prominent clinical value during radiotherapy planning [58,59]. The application of nanoparticles could scavenge free radicals to protect normal tissues. Radioprotector molecules e.g., flavonoids encapsulated in nanomaterials such as liposomes, fullenes, and polysaccharides, can be used effectively during RT [60,61,62].

Nanoparticles can enhance radiosensitivity due to stronger photoelectric absorbance capacity [8,63,64]. Nanoparticles can promote the immune responses along with radiotherapy in combinatorial regimens with anti-PD1 blockade [65]. The idea of stimulating systemic immunotherapy effects with local treatment of radiotherapy is an effective strategy for the development of next generation cancer radioimmunotherapy, especially for comprehensive treatment and management of advanced cancers which cannot be cured by conventional therapeutic strategies.

## 4. Experimental Studies on Nanoparticles Combined with Radiotherapy

### 4.1. Nanoparticles Carrying Heavy Elements for Enhancing Cytotoxic Effects of Radiotherapy via High-Z Effect

**Figure 1:**
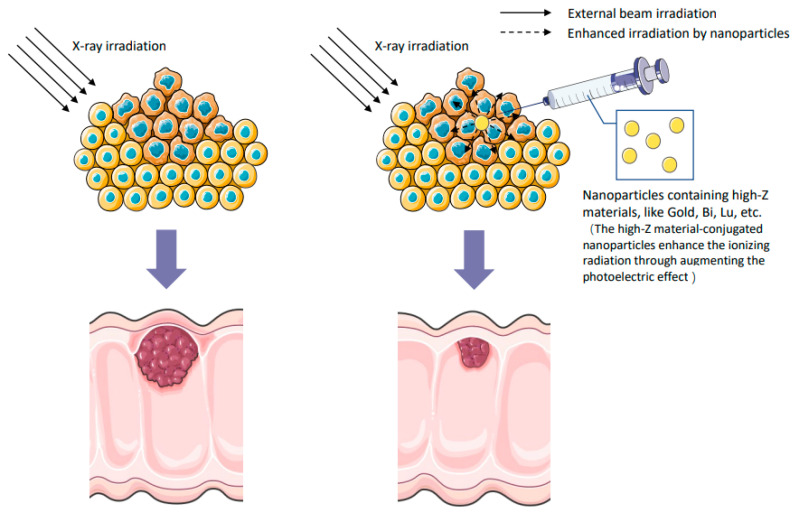
Comparative schematic depiction of implications of high Z-effect metal (Au, Bi, Lu) nanoparticles containing radiosensitizing drug moieties for alleviating tumor growth through enhancing the X-ray irradiation-induced damage when compared to radiation therapy alone. The high-Z material-conjugated nanoparticles enhance the ionizing radiation through augmenting the photoelectric effect.

Many researchers have investigated several nanoparticles for improving the cytotoxic effect of radiotherapy and inhibiting the systemic progression of locally advanced tumors. The nanoparticles carrying high-Z elements are the most widely explored in radiation therapy against tumor cells (Figure 1). High-Z nanomaterials can assimilate, scatter, and eradicate irradiation energy due to low-energy photoelectrons and auger electrons [66].

Dose partitioning is a profound effect observed when nanoparticles locate around the tissue when radiotherapy irradiates onto tumor tissue. A significant difference in the atomic mass of nanoparticles when compared to average atomic numbers in cells of ≈3–7 is evidently observed for high Z nanoparticles [35]. During this therapy, the released photoelectrons and auger electrons can penetrate into the tumor cells and hydrolyze the H_2_O molecules to generate free radicals, which can ultimately lead to DNA double strand breakage [67,68]. For high-Z nanomaterials, the probability of interaction is usually quantified as the total mass attenuation coefficient (μ/ρ), representing the probability of interaction per mass unit of a specific material (in cm^2^/g). For instance, the ‘photoelectric effect of gold’ is significantly stronger at energies of 200 keV and below. Consequently, gold attenuates more energy per gram than water for low-energy photons especially for those used in low-dose-rate brachytherapy. However, it is widely reported that substantial radiosensitization effects can be observed at much lower gold concentrations which significantly increase the dose in the medium; this concludes the intricate role of biological and chemical interactions of gold nanoparticles in radiosensitization [69].

#### 4.1.1. Au

Gold nanoparticles (GNPs) have been one of the most widely explored materials for several years for the reinforcement of radiation dose effects across the tumor volume [70]. This “gold nanoparticle-assisted radiation therapy” was primarily proposed in the year 2009 by Cho et al. [71,72], and was verified and explored in subsequent research studies [73,74,75,76,77]. Au nanoparticles exhibit surface plasmon resonance and photothermal properties, which lead to diagnostic and therapeutic efficacy [78]. The therapeutic efficacy of GNPs in combination with radiotherapy can be observed evidently due to their well-defined parameters in terms of size, shape, and surface chemical properties [17,79,80]. The effective size range is smaller than 50 nm for therapeutic efficacy, and those with a size range of 2 to 3 nm are especially effective, which is crucial for enhancing dose deposition [81]. Zhuang M. et al. used GNPs loaded with small interfering RNA (siRNA)-SP1 (AuNPs-si-SP1) for inducing radiosensitization during radiotherapy against lung cancer. Au nanoparticles (GNPs) in this conjugate are efficient in augmenting radiosensitivity in lung cancer cells, which is accompanied by mitigated cell viability which subsequently fosters cell cycle arrest and induces programmed cell death [82]. Furthermore, the loaded siRNA in AuNPs-si-SP1 also shows a significantly better performance in suppressing SP1 mRNA and protein expression in A549 lung cancer cells when compared to the free siRNA-SP1 [82,83]. AuNPs-si-SP1 in tumor-bearing mice models can significantly impair the proliferation of cancer cells in solid tumors via inhibiting SP1, and upregulates granzyme B (GZMB) [82]. Chiang C. et al. have explored ‘Au nanoparticles’ in the tumor cell-targeting radiotherapy of glioblastoma multiforme (GBM), a malignant tumor with a low survival rate of 10 to 14 months after diagnosis [84]. They synthesized a Golden Disk nanoplatform as a radio-enhancer (Hac-Au@SiO_2_). Intracellular accumulation of Hac-Au@SiO_2_ at different time points (3 h, 8 h, 24 h, and 72 h after incubation) showed that the nanoparticle can impair CD44 expression on GBM cells to perform tumor cell-targeted radiotherapy [84]. Experiments on tumor-bearing nude mice have also confirmed the implications of Hac-Au@SiO_2_ in combination with low-dose RT (2 Gy) for enhancing safety and therapeutic efficacy; therefore, this ‘nanoparticles administration in combinatorial regimen with radiotherapy’ tends to be a more promising candidate in clinical settings for improving clinical outcomes in cancer patients [85].

The introduction of nanoparticles helps tumor cell-targeted radiotherapy for some malignancies such as breast cancers due to their intricate molecular pathophysiology. For instance, nanoparticle implications during radiotherapy are considered as a significant topic to explore the improved therapeutic effects against triple negative breast cancer (TNBC) due to deficient expression of ER, PR or HER2 [38,86,87,88,89]. The expression of CXCR4 chemokine receptor is observed among all the molecular subtypes of breast cancers and the upregulation of CXCR4 is significantly correlated with a higher incidence of lymph node metastasis, desmoplasia, and mitigated T-lymphocyte infiltration [90,91,92]. Bhattarai S. et al. used gold nanoparticles to target these receptors via conjugating the anti-CXCR4 antibody (cGNPs). They evaluated the ionizing radiation level in the cGNPs-treated tumor cells after receiving radiotherapy. The cGNPs treatment showed a higher specific intracellular concentration in breast cancer cell lines with X-ray irradiation. Furthermore, the irradiation-induced ROS generation and DNA double strand breaks were higher in the cells with CXCR4 receptor overexpression when the radiotherapy was given in combination with cGNPs. The cGNPs treatment in combination with RT also resulted in tumor shrinking by blocking their regrowth even after 50 days, which suggested therapeutic improvements in radiotherapy [93].

#### 4.1.2. Bi

In addition to the high-Z metals, some semiconductors have also shown high-Z characteristics, and can therefore act as radiosensitizers [91,94,95]. In recent years, many researchers have described the implications of high-Z semiconductor nanoparticles in radiotherapy due to their versatile physicochemical properties [96,97,98,99]. Zang Y. et al. showed that high-Z Bi_2_WO_6_ semiconductor materials had good solubility and biocompatibility; these materials exhibit significant potential to serve as contrast media and radiosensitizers for improved imaging and radiotherapy efficacy, respectively. A combinatorial regimen of Bi_2_WO_6_ nanoparticles with X-ray irradiation exhibited significant impairment of tumor growth in vitro and in vivo models. Toxicity analysis was performed using blood and serum obtained from the in vivo mice models which received the above regimen of Bi_2_WO_6_ nanoparticles and radiotherapy [100]. There was no toxicity profile reported with this therapy [100]. Cheng X. et al. also formulated Bi_2_S_3_ nanorods to act as a contrast medium for computed tomography (CT) as well as a radiosensitizer. In addition to the X-ray attenuation property via high-Z effects, Bi_2_S_3_ nanorods can be effective to mitigate invasion and metastasis of tumor cells in vitro/in vivo models. Furthermore, nanomaterial-mediated hyperthermia can enhance oxygen tension in hypoxic areas in solid tumors and impair HIF-1α expression to overcome the radioresistance [101].

#### 4.1.3. Lu

Jeroen G. et al. described the importance of endoradiotherapy with ^177^Lu-labelled star polymers as a promising novel therapeutic approach against tumor cells due to their enhanced permeability and retention (EPR) characteristics. Mice bearing subcutaneous CT26 isografts (an example of a high EPR tumor) received a high dose of ^177^Lu-labelled star polymers (7.4 MBq) and this treatment significantly prolonged survival rates in the mice. In this study, the inhibition of tumor growth was positively correlated to the therapy doses. However, there were no significant changes reported pertinent to the blood markers except a decline in white blood cells caused by endoradiotherapy; this decline is often counteracted by granulocyte colony stimulating factor (G-CSF) in clinical settings. The multi-measured biodistribution profiles of ^177^Lu-labelled nanoparticles also demonstrate a remarkably higher uptake of these nanoparticles into the tumor tissues and low accumulation in other organs [102]. As we discussed above, Lin M. et al. conjugated the β-emitter ^177^Lu to DTPA-polyethylene glycol (PEG) decorated gold nanostars (^177^Lu-DTPA-pAuNS) and explored it as a potential radiopharmaceutical agent for head and neck squamous cell carcinoma (HNSCC) [39]. The in vivo and in vitro accumulation, therapeutic efficacy and the enhancement effects of ^177^Lu-DTPA-pAuNS were compared and verified on orthotopic HNSCC tumor models and cell lines, respectively. The survival rates among different treatments were also compared using the Kaplan–Meier method [39].

### 4.2. Nanoparticles Enhanced Radiotherapy Efficacy via Alleviating Tumor Hypoxia

**Figure 2:**
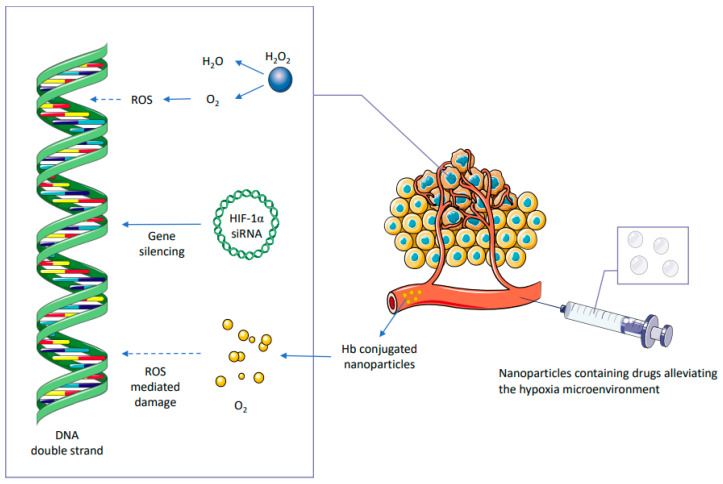
The implications of nanoparticles containing radiosensitizing drug moieties for alleviating tumor hypoxia. For instance, Hb-conjugated nanoparticles could induce ROS-mediated DNA damage through enhancing oxygen tension. In addition, HIF-1α expression could be downregulated using siRNA-mediated gene silencing using nanocarriers during radiotherapy or brachytherapy, which subsequently fosters radiosensitization.

Tumor recurrence and progression during radiotherapy are due to the radioresistance produced by tumor cells [103,104,105]. Radioresistance across tumor hypoxia is a prominent hindrance for the improvement of clinical outcomes with radiotherapy in cancer patients [106,107,108]. Limited angiogenesis can cause limited oxygen diffusion across the tumor microenvironment, which often leads to widespread tumor hypoxia and subsequently contributes to the reversal of radiation-induced DNA damage and subsequent induced high tumor cell proliferation (Figure 2) [109,110]. In addition, oxygen is considered as a radiosensitizer. The tumor regions seldom exhibit anoxic environments and tumor hypoxia could induce mitigation of ROS production as well as oxygen-fixation reaction, subsequently causing low efficacy of radiotherapy [67,103,111,112,113]. Activation of HIF-1α during hypoxia results in the higher gene expression pertinent to angiogenesis, invasion, and metastasis and subsequently fosters chemoresistance and radioresistance [114,115,116]. Upregulation of HIF-1α is evident during hypoxic environments through the HIF prolyl hydroxylases [117,118]. Oxygen is required for the activity of these enzymes during HIF degradation; thus, the inactivation of prolyl hydroxylases results in the accumulation of HIF. On the other hand, HIF activation is also mediated via unfolded protein response (UPR) and mTOR signaling [118]. HIF-1α induces gene expression and acquisition of tumor cell adaptation in hypoxic environments by mitigating oxygen consumption, thus shifting the cells towards a glycolytic pathway which requires lower oxygen levels than oxidative phosphorylation [119]. Furthermore, HIF-1α can enhance angiogenesis and subsequently fosters blood flow into the tumor hypoxic microenvironment [120]. A higher glycolytic metabolism results in higher lactate generation which subsequently induces acidification of the extracellular environment [121]. Hence, it is crucial to use radiosensitizers specific for hypoxia through non-invasive imaging methods during radiotherapy against tumor-hypoxic regions [122]. For instance, as we discussed above, the administration of GNPs in a combinatorial regimen with radiotherapy could enhance the anti-tumor therapeutic efficacy across hypoxic tumor regions [122].

Hypoxic regions across tumors could serve as ideal environments for anaerobic bacteria; recent studies report the application of bacteria for selective targeting of hypoxia or anoxia tumor regions, which subsequently activate immune responses [123,124,125]. The radiation-induced ROS production is constrained by tumor hypoxia due to insufficient blood flow in animal tumor models. This condition was explained by the hyperbaric oxygen in clinical settings by using blood substitutes which carry oxygen or hypoxic radiosensitizers, including misonidazole, metronidazole, and tirapazamine. These strategies can effectively impair growth of hypoxic tumor cells [126,127]. Nitroimidazole is specifically used as an imaging drug and it can modulate tumor hypoxia, subsequently ameliorating radioresistance induced through hypoxia by sensitizing tumors to radiotherapy-induced ROS production [128]. In addition, smart ‘nanogels’ are impregnated with a nitroimidazole derivative i.e., iodoazomycin arabinoside in combination with galactose. These are significantly used for the sensitization of liver cancer cells in hypoxia conditions [128]. Another study proved the efficacy of nanoparticles impregnated with metronidazole and temozolomide (DNA alkylating agent) to target glioblastoma [129]. This combinatorial regimen enhanced the survival rate in in vivo mice models compared to the mice which received radiation therapy alone [129]. Liposomal pimonidazole is another drug formulated in nanoparticles and administered along with radiotherapy to induce sensitization of melanoma cells in hypoxia conditions [130]. Perflurohexane impregnated with liposome formulations can have a higher oxygen capacity and it can foster direct oxygen supply to the tumor hypoxia. In one study, the combination of nanoparticles and radiotherapy mitigated tumor growth more than the individual regimen of radiotherapy in a mouse model [131]. Nanoformulations of dodecafluoropentane are used to enhance oxygen supply in tumor hypoxia conditions in vivo mouse models, which consequently enhances the responses of radiation therapy [132].

As we discussed above, for example, hafnium oxide-loaded nanoparticles (NPs) and PEGylated gold or manganese dioxide NPs have been significantly explored in preclinical settings to enhance radiosensitization. A recent study showed that the administration of manganese dioxide NPs in the hypoxic murine or human xenograft breast tumor models enhanced the efficacy of radiation therapy and subsequently mitigated tumor progression, growth, and expression of angiogenesis markers such as VEGF [133]. Another study described the significant benefits of attenuated strains of *Salmonella typi* Ty21A, anaerobic bacterium as radiosensitizers and as carriers to deliver GNPs across the hypoxic tumor volume. According to this study, several kinds of modified nanoformulations such as ‘Citrate-GNPs, Gelatin-GNPs, BSA-GNPs, FA-GNPs, Glutamine-citrate-GNPs, Glut-BSA-GNPs, Glu-BSA-GNPs’ by *Salmonella typhi* Ty21a were studied in combination with radiotherapy. Among these, the FA-GNPs are predominantly considered as the best choice for the preparation of golden bacteria which can induce a higher delivery of GNPs across anoxic tumor volume. Subsequently, this strategy of delivery mitigated the radioresistance across the tumor microenvironment. The bacteria-mediated nanoparticle delivery exhibits a significant photothermal therapy across the tumor volume [123]. The in vivo biodistribution of NPs has a significant influence on the efficacy of radiotherapy and tumor hypoxia; this biodistribution solely relies upon several factors such as size, shape, charge, and other surface properties [17].

In addition to the aforementioned high-Z effect, GNPs have been applied for enhancing the generation of ROS-mediated oxidative stress [134,135,136]. It is well acknowledged that ROS are closely related to programmed cell death and necrosis [137]. The synthesized α-Fe_2_O_3_@Au nanoparticles have shown significant efficacy in enhancing ROS production. Radiation-induced ROS generation in combination with α-Fe_2_O_3_@Au nanoparticles was significantly higher when compared to individual treatment groups. Assessment of intracellular DNA damage by the formation of γ-H2AX foci also showed detectable γ-H2AX foci in the combinatorial regimen of the “α-Fe_2_O_3_@Au and X-ray” group [138]. Emerging research reports suggested that the delivery of hypoxia-responsive prodrugs (tirapazamine (TPZ)) encapsulated in nanocarriers can play an effective role in modulating the hypoxia condition in tumor tissues when combined with radiotherapy. TPZ is an anti-tumor agent which shows toxicity to the tumor cells at relatively low oxygen tensions [139]. A multifunctional nanoradiosensitizer (TPZ@UCHMs) induced tumor targeting efficacy and this was successfully synthesized to encapsulate the TPZ to overcome the oxygen dependency during radiotherapy. The efficacy of these nanoparticles was evaluated in vitro and in vivo models to explore the modulating effect of TPZ on radiosensitivity. The role of oxygen and TPZ@UCHMs in radiation-induced cytotoxicity was ascertained by a colony-forming assay [140]. The survival fraction of cancer cells treated with TPZ@UCHMs in combination with RT significantly decreased when compared to hypoxic cells co-cultured with individual treatment groups of TPZ or UCHMs. The therapeutic efficacy of the combination of RT and TPZ@UCHMs was observed through the inhibition of hypoxia and their counteracting ability against hypoxia-mediated metastasis in tumor-bearing nude mice [141].

Recent research studies have described the efficacy of hemoglobin (Hb)-based oxygen carriers for ameliorating tumor hypoxia, as these carriers exhibit substantial oxygen-loading capacity [142]. Despite the fact that Hb can be employed as an efficient component for oxygen delivery, its usage is constrained by nephrotoxicity and immunogenicity [143]. In order to overcome this obstacle, Xia D. et al. synthesized Au-Hb@PLT to explore its radiosensitization efficacy; Au-Hb@PLT alleviated hypoxia in tumor tissue through adequate oxygen delivery during radiotherapy [144]. Au-Hb@PLT could directly deliver the oxygen molecules and acted as an effective radiosensitizer across the tumor tissues, subsequently enhancing clinical outcomes during radiotherapy. Tumor targeting efficacy of Au-Hb@PLT when combined with nanoparticles was evaluated in HeLa cells and tumor-bearing nude mice [144]. Similarly, Sang W. et al. evaluated the efficacy of nanoparticles encapsulated (Hb@Hf-Ce6) with Hb molecules to reduce the toxicity and immunogenicity without impairing the ‘oxygen delivering function’ [145]. In addition to the oxygen-delivering effect of Hb, a higher intracellular ROS generation in 4T1 cells treated with Hb@Hf-Ce6 was also observed during X-ray irradiation; subsequently, this ROS-mediated oxidative stress was predominantly higher due to high-Z metal hafnium (Hf), which enhances DNA damage induced by radiotherapy [145]. The pharmacokinetic properties and anti-tumor effects of nanoparticles encapsulated with Hb molecules when combined with the ‘X-ray irradiation’ and ‘PD-1 checkpoint blockers’ in metastatic tumor models showed that application of a nanoformulation strategy could be an effective strategy to ameliorate tumor hypoxia and to enhance radiosensitization [146].

H_2_O_2_, an abundant metabolite in tumor tissues, plays a crucial role in maintaining a malignant tumor phenotype and activating HIF-1α expression, which can aggravate the hypoxia-induced tumor resistance [147,148]. In addition, catalytic decomposition of H_2_O_2_ can be considered as an ideal strategy for enhancing the efficacy of radiotherapy and relieving the hypoxia-induced radioresistance [149,150]. Chen Y. et al. formulated a catalase-like MOF-based nanosensitizer (MnTCPP-Hf-FA MOF NPs) along with radiotherapy in order to target tumor cells. The fluorescence probe MI-H_2_O_2_ was used to ascertain the catalase-like activity of the nanosensitizer. Elevated O_2_ tension and degradation of H_2_O_2_ inside the tumor microenvironment suggested a higher efficacy of MnTCPP-Hf-FA MOF NPs. Additionally, the nanoparticle-mediated ROS generation along with X-ray irradiation was also verified by the singlet oxygen sensor green agent [151]. Subsequently, xenograft melanoma tumor models were further used to examine the therapeutic efficacy of these NPs in nude mice. The results of this study showed that the evident inhibition efficacy of tumor growth was observed with a combinatorial regimen of nanoparticles and RT [152].

As we discussed above, HIF-1 can also mediate tumor hypoxia [153,154,155,156,157,158]. The HIF-1 protein relies mainly on two subunits, named HIF-1α and HIF-1β, to modulate the O_2_ homeostasis via increasing the O_2_ availability and promoting adaptation of tumor cells to O_2_ deprivation [159]. Several studies have reported that HIF-1 is overexpressed in different cancer types, which plays an important role in activating transcription enzymes and proteins associated with tumor proliferation, invasion, and chemo-/radioresistance under hypoxic conditions [160,161]. Therefore, many therapeutic strategies have focused on regulating HIF-1 gene expression, especially in combination with radiotherapy. For instance, HIF-1 inhibitors can effectively downregulate HIF-1 gene expression [162]. In one study, the nanomaterial was encapsulated along with HIF-1 inhibitors to improve their pharmacokinetic characteristics. Zhou X. et al. explored the possibility to utilize Cu_2-χ_Se@PtSe (CSP) nanoparticles functionalized with the HIF-1α inhibitor acriflavine (ACF) to enhance radiosensitivity [163]. The results of this study suggested that the hypoxic environment was significantly alleviated via H_2_O_2_ decomposition in the treatment group receiving CSP-ACF in combination with RT (10 mg/kg, 6 Gy) [163]. The HIF-1 gene expression was also evaluated in this study to confirm that this expression cannot be efficiently repressed by irradiation alone when compared to the combination regimen of CSP-ACF and X-rays [163]. Interestingly, another important indicator, VEGF, an angiogenesis and tumor growth factor, was also downregulated during the combinatorial regimen of CSP-ACF with X-ray irradiation compared to other individual treatment groups; this indicates that the angiogenesis of tumor microvessels tends to become less dense after the treatment of CSP-ACF nanoparticles combined with radiotherapy [163]. With respect to tumor angiogenesis, recent research reports have unveiled a higher post-radiation vascular reconstruction in tumor microenvironments by HIF-1 activity [119,164], which shows a profound efficacy of anti-angiogenesis therapy along with radiotherapy, which effectively targets HIF-1 gene expression in hypoxic tumors. Apart from nanoconjugated drugs, other nanocarriers have been investigated as smart delivery systems for transporting ‘HIF-1 targeting siRNAs’ into hypoxic tumor cells. Calcium phosphate (CaP)-encapsulated nanoparticles are effective for siRNA delivery, as the formulated nanoparticles can undergo dissolution in the low pH microenvironment and deliberately release the encapsulated siRNA into the cytoplasm [165,166,167,168]. Chen W. et al. used ‘anisamide targeted lipid-calcium-phosphate (LCP)’ nanoparticles to deliver siRNA for targeting the HIF-1α into the ‘tumor cell cytoplasm’ combined with photodynamic therapy. HIF-1α encapsulated with siRNA in this nanoformulation can mitigate the expression of HIF-1α, subsequently promoting cell death [169]. Similarly, nanoparticles can be applied for the effective inhibition of the signaling axis involving HIF-1α in order to suppress tumor growth [170,171].

A Few examples of studies which delineated the efficacy of nanoparticles-formulations to improve efficacy of radiotherapy through in vitro/in vivo models of cancers were given in (Table 1).

### 4.3. Nanoparticle-Enhanced Radiotherapy Efficacy via the Deliberate Delivery of Drugs and Other Functional Anticancer Molecules

Apart from the aforementioned mechanisms, nanoparticles can bind to the functional molecules associated with targeting specific signaling proteins involved in cancer progression. During radiotherapy, intracellular oxidative stress is induced by ROS generation, which further fosters cytotoxicity [174,175]. The tumor cells induce counter signaling cascades to neutralize intracellular ROS through antioxidant mechanisms (for example, overexpression of glutathione (GSH)) [176]. Dong Z. et al. prepared a gallic acid-ferrous (GA-Fe(II)) nanocomplex for mediating Fenton reaction and this complex can enhance the production of highly cytotoxic hydroxyl radicals (•OH) when encapsulated with GA-Fe(II) and an ‘inhibitor of GSH synthesis’ (L-buthionine sulfoximine, BSO) into a stealth liposomal nanocarrier (BSO/GA-Fe(II)@liposome). The effects of this nanocomplex for anticancer efficacy were examined in in vitro models of 4T1 tumor cells and in vivo models of tumor-bearing nude mice. Combination therapy of BSO/GA-Fe(II)@liposome and X-ray irradiation effectively impaired the 4T1 cancer cell growth and in vivo tumor models when compared to the individual treatment groups of nanoparticles or X-rays. Furthermore, the blood analysis and serum biochemistry indexes of the blood derived from nude mice treated with above BSO/GA-Fe(II)@liposome formulation showed a significantly higher anti-tumor efficacy of liposome-formulated chemotherapy and radiotherapy when compared to the individual treatment groups or control group with nanoparticle injection alone. Thus, adjuvant BSO/GA-Fe(II)@liposome-encapsulated nanomedicine fostered substantial anti-tumor efficacy with minimal side effects on the normal tissues [172].

DNA repair in the tumor cells is mediated by poly (ADP-ribose) polymerase (PARP). Hence, PARP inhibitors could impair DNA repair and enhance the formation of broken DNA strands [177,178,179]. In addition, treatment with PARP inhibitors can cause breakage at the replication fork which consequently reduces the efficiency of radiation therapy [177,178]. Olaparib is a PARP inhibitor which can induce the formation of broken DNA strands and olaparib combinatorial regimen with RT fostered the efficacy of RT in both in vitro and in vivo studies [180,181,182,183]. However, olaparib is associated with poor solubility and higher toxicity, which limits its usage in the clinical setting. Therefore, the application of nanosystems for the formulation of olaparib is a preferred strategy to enhance the targeting efficiency against tumor growth [184,185,186]. Overexpression of folate receptors is reported in tumor cells of cervical cancer, ovarian cancer, and nasopharyngeal cancer [187]. Folate receptors could be effective oncotargets due to their differential expression in cancer cells [188]. Nanoformulation systems conjugated with folate can target folate receptors, and these nanodelivery systems can be considered as significant anticancer drug carriers [189,190]. Olaparib-encapsulated nanoparticles can improve radiosensitivity in xenograft mice models of lung cancer [191]. Application of ‘folate-conjugated honokiol nanoparticles’ bestows active targeting efficacy in xenograft mice models of nasopharyngeal cancer [183,191,192].

**Figure 3:**
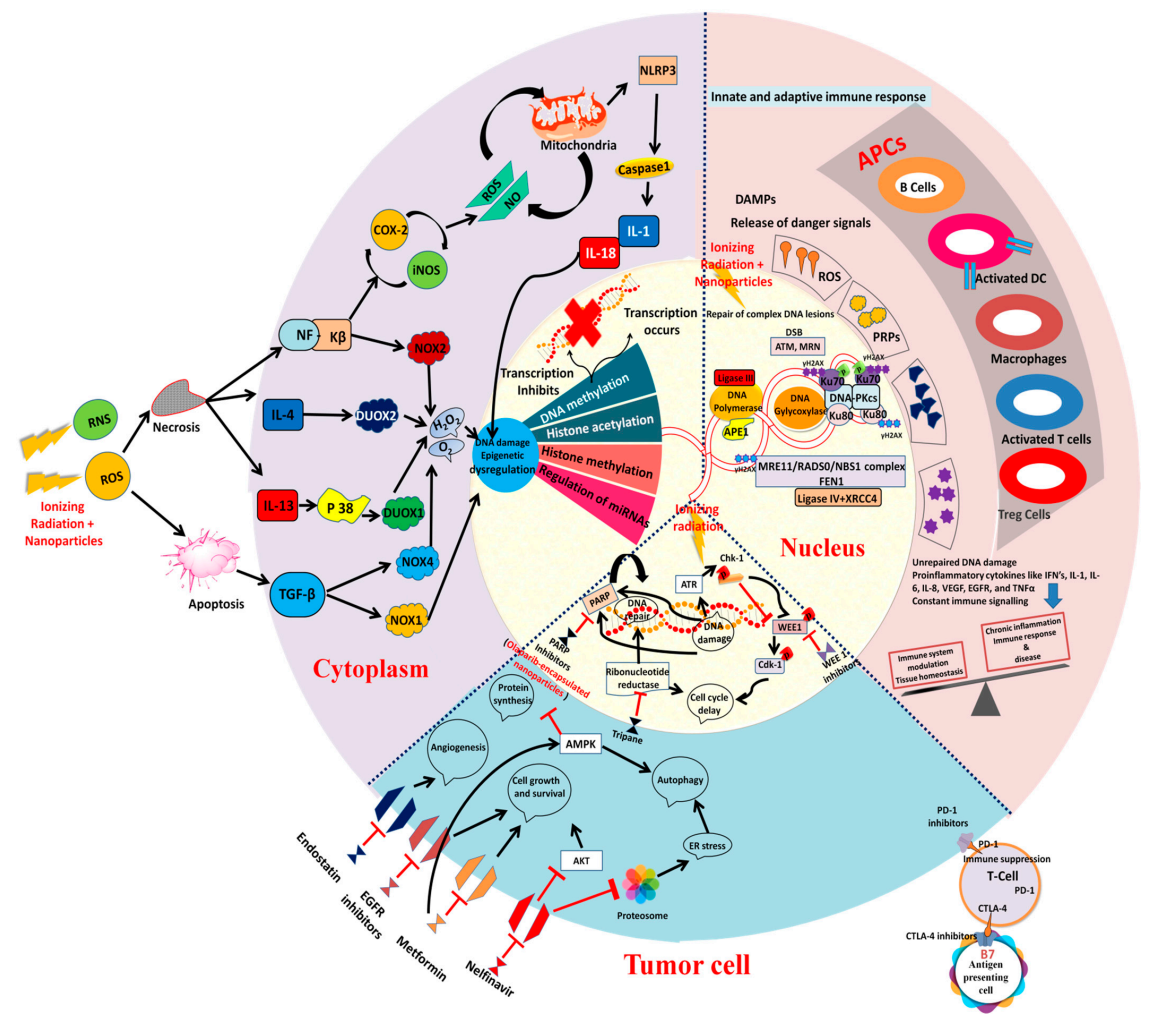
Schematic depiction of the efficacy of nanoparticle formulation in combination with ionizing radiation for inducing (1) necrosis and apoptotic mode of tumor cell death by inducing epigenetic alterations (?) and DNA damage through the generation of mitochondria-mediated ROS (reactive oxygen species), RNS (reactive nitrogen species); (2) Olaparib-encapsulated nanoparticles in a combinatorial regimen with radiation could induce inactive PARP and consequently foster a delay in the cell cycle and induce DNA damage. In addition, adjuvant therapies such as EGFR blockers, metformin, nelfinavir or endostatin could block the cell growth, cell survival, Akt protein, and angiogenesis and subsequently tumor cells acquire autophagy mode of cell death; combination of radiotherapy and anti-PD-1/PD-L1 therapy could be effective in metastatic tumors; (3). Nanoparticles as an adjuvant to radiation therapy could induce enhanced formation of DSB (double stranded DNA breaks) and cause unrepaired DNA damage. ROS, PRPs (platelet rich plasma factors), Systemic immune effects are also produced which results in the immune system modulation. (?): requires study.

Olaparib-encapsulated nanoparticles prevent PARP release during radiation therapy-induced DNA damage, which consequently amplifies the effect of RT [173]. This kind of radiosensitization of olaparib nanoparticles has proven effective in prolonging median overall survival when compared to the individual treatment groups of RT or olaparib nanoparticles in nude mice models of cervical tumor models [173] (Figure 3).

## 5. The Implications of Nanoparticles in Preclinical/Clinical Studies in the Field of Radiation Oncology

In recent years, malignancy still constitutes a considerable proportion of cancers, which leads to poor overall survival [193]. Hence, technical advances in effective conventional cancer therapy management are considered as an urgent need to improve overall survival and quality of life in clinical settings [194,195]. Viral vectors, lipid-based nanocarriers, and inorganic nanoparticles are prominent nanomaterials applied in clinical settings [196,197,198]. However, the progress in clinical transformation of nanoparticles for targeting tumors during radiotherapy is relatively slow, which tends to be a further opportunity for future investigation [199]. We here discuss research pertinent to the nanoparticle technology which can be applied in clinical trials of radiotherapy.

Hafnium oxide nanoparticles (NBTXR3) are radioenhancers widely explored through experimental studies; these nanoparticles could enhance the efficacy of X-ray irradiation and alleviate the cancer cell proliferation and distant metastasis of malignant tumor cells. NBTXR3 induces energy dose deposit through high electron density and fosters the immune system priming dependent on CD8+ lymphocyte T cells [200,201,202]. Injection of NBTXR3 for patients diagnosed with locally advanced soft-tissue sarcoma in a multicenter, randomized controlled phase I/II clinical trial (NCT02379845) demonstrated significant improvements in the pathological complete response (pCR) or objective response rate (ORR) [203]. A total of 176 patients diagnosed with locally advanced soft-tissue sarcoma were randomly divided into ‘NBTXR3 group’ receiving NBTXR3 activated by external-beam radiotherapy, (*n* = 87), and ‘control group’ receiving RT only (*n* = 89) [203]. A total of 14 (16%) out of 87 patients in the NBTXR3 group achieved the primary endpoint (pCR, <5% of residual viable tumor cells) compared to 7 (8%) out of 89 patients in the radiotherapy group [203]. The key secondary endpoint pertinent to the resection margin after neoadjuvant treatment suggested that more patients in the NBTXR3 group achieved R0 margins compared to RT group [203]. The adverse events related to NBTXR3 typically lasted for a short duration, but these adverse effects were easily resolved immediately in the majority of the patient cases [203]. A previous phase I trial on NBTXR3 was conducted on 22 patients diagnosed with locally advanced soft-tissue sarcomas who received a single injection of NBTXR3, external beam radiotherapy (EBRT) and tumorectomy. In this study, the patients did not exhibit any local recurrence, with only five patients reporting distant recurrence [204].

The ^177^Lu-DKFZ-PSMA radioligand is preferred as a novel theranostic agent during radiotherapy due to higher efficacy and safety. This agent exhibited a significantly higher accumulation in prostate-specific membrane antigen (PSA)-positive tumors and was proven to be highly effective in fostering radiotherapeutic efficacy in preclinical studies [205,206,207]. Recently, this therapeutic strategy was used against metastatic castration-resistant prostate cancer (mCRPC) in preclinical trials. In an interventional clinical trial, a total of 31 patients with prostate cancer diagnosed with local/nodal/distant metastatic tumors received ^177^Lu-DKFZ-PSMA-617 therapy. After 3 months of follow-up, the biochemical, metabolic, and clinical responses were evaluated at three different time points which included 2 weeks, 1 month, and 3 months after each therapeutic cycle [208]. A significant improvement in the therapeutic response or overall survival was observed with ^177^Lu-DKFZ-PSMA-617 therapy compared to the patients who did not receive this therapy [208]. The median overall survival (OS) and progression-free survival in this study were 16 months and 12 months, respectively. Life-threatening toxicity was not observed in these patients, concluding that ^177^Lu-DKFZ-PSMA-617 therapy is a safe and effective therapeutic option for the treatment of terminal mCRPC patients [208]. Similar anti-tumor efficacy was reported in subsequent clinical trials [209,210]. In another dual-center phase II clinical trial, a total of 47 patients diagnosed with progressive metastatic CRPC received ‘lutetium-177-labeled anti-PSMA monoclonal antibody J591 (^177^Lu-J591) therapy’ after hormonal therapies. The median OS for all the patients in this study was 17.6 months (95% CI, 15.2–20 months), with improved survival for the high-dose group (70 mCi/m^2^, median OS = 21.8 months [95% CI, 16.3–27.3 months]) when compared to the low-dose group (65 mCi/m^2^, median OS = 11.9 months [95% CI, 6.5–17.3 months], *p* = 0.03), respectively. However, the patients experienced hematologic toxicity at a median of 4 weeks after ^177^Lu-J591 application, which was mainly in the form of grade-4 thrombocytopenia lasting a median of 7 days, without the incidence of recurrent hemorrhagic episodes [211].

In a dual-center, single-arm clinical trial, a total of 66 patients pathologically diagnosed with glioblastoma (among them 59 with recurrent glioblastoma) received neuronavigational instillation of iron oxide nanoparticles in the form of an aqueous dispersion through intratumoral route [212]. Stereotactic external radiation dosage of 30 Gy was administered in the planning target volume, fractionated as 5 × 2 Gy per week; this radiation dose was given immediately before or after the activation of nanoparticles via intratumoral thermotherapy [212]. The median OS from the period of diagnosis of the first tumor recurrence among 59 patients with recurrent glioblastoma was 13.4 months (95% CI: 10.6–16.2 months). The median time interval between primary diagnosis and first tumor recurrence was reported as 8 months. However, there were no prolonged side effects observed except minor hemiparesis due to the primary disease [212]. However, this study has a few drawbacks. Primarily, the metal materials within 40 cm of the treatment area need to be removed, and the indefinite exclusion of MRI for subsequent diagnosis of tumor progression is also an obstacle [212].

In another phase I dose-escalation study on patients with multiple brain metastases, theranostic activation and guidance of irradiation by X-ray (AGuIX) nanoparticles’ was conducted for radiosensitization and as a magnetic resonance imaging (MRI) contrast [213]. Preclinical studies revealed that AGuIXis considered a non-toxic radiosensitizer with significant therapeutic effects at minimal concentrations [214,215,216,217]. A total of 15 patients diagnosed with multiple brain metastases received ‘AGuIX nanoparticles’ in combination with ‘whole brain RT (WBRT)’ in this prospective phase Ib dose-escalation clinical trial [213]. Tolerability, therapeutic effects, and adverse events for the AGuIX nanoparticles were evaluated at different time points before and after treatment. The treatment of theranostic AGuIX nanoparticles significantly mediated amelioration of multiple brain metastases [213].

Most of the recent research on palladium has concentrated on the therapeutic effects and clinical benefits of palladium as a radiation source (for instance, palladium-103 radiotherapy) [218]. Palladium nanosheets enhance the efficacy of radiation therapy through radiosensitization of tumor cells to therapy; these nanosystems exhibit sheet-like characteristics such as ‘diameter of ~14 nm and a thickness of ~2 nm’. The usage of these nanosheets could promote anti-tumor efficacy even at lower X-ray doses [218]. In the future, research studies should explore the possibility of palladium as a nanomaterial component of noble metal-based NPs to enhance the efficacy and clinical benefit of tumor radiotherapy. Thus, the clinical translation of encapsulated nanoparticles in combination with radiation therapy will deliver effective radiosensitization for conventional fractionated RT. In addition, studies related to external-beam radiotherapy, as in radioimmunotherapy, or implanted radioactive components as specific energy sources, as in brachytherapy, have to be extrapolated to other tumor types for clinical application [35].

## 6. Current Limitations and Challenges: Nanoparticle Applications in the Field of Radiation Oncology

Despite the continuous exploration of cutting-edge technologies, the field of clinical oncology treatment is relatively slow. The administration of nanoparticles to improve the therapeutic efficacy of radiotherapy to target tumors still faces several challenges and obstacles. Only a few classes of nanoparticles have gained importance, predominantly in preclinical studies; subsequently, they are currently in clinical trials (Table 2).

Firstly, the unique properties of nanomaterials are quite different from traditional materials in terms of the structure, composition, size, surface properties, charge, and aggregation behavior; these properties could make their formulations harder to translate into the clinical setting through conventional processing techniques [237,238]. Therefore, nanoparticles should be processed on a batch-to-batch basis with the aid of several novel techniques. Nanocarriers can effectively interact with biological fluids such as blood, serum, and intracellular fluid [239], which further modulate the function of nanoparticle components in biological systems [240,241]. The half-life and stability of nanoparticles also limit their clinical use [242]. Biodegradable nanoparticles have gained importance in cutting-edge research for cancer treatment [243]. Furthermore, the administration of clinically relevant nanoparticles into cancer patients is constrained due to their safety and toxicity. Nanoparticles may be associated with unfavorable biological interactions which need further scientific exploration, i.e., nanotoxicology [244,245,246]. In order to introduce nanotechnology into clinical settings in a safe way, the therapeutic effects and risks associated with nanomaterials should be appropriately optimized by combining more advanced targeting strategies [247,248,249,250,251,252]. Finally, robust supervisory policies and stringent regulations are needed for the efficient development of nanoparticle technology in clinical radiotherapy [253,254]. Regulation should not only be associated with relevant clinical trials, but is also a vital part of the development of novel technologies [255,256,257], specifically for the characterization and quality control of nanomaterials [258].

## 7. Conclusions

The application of nanomaterials is a novel approach to induce radiosensitization during clinical radiation therapy and is well tolerated at low doses. Engineered nanomaterials in a perfect state can enhance the effects of radiation therapy by enhancing tumor selectivity and mitigating side effects. In addition, recent breakthroughs in nanotechnology facilitate effective molecular targeting and gene/drug delivery. Nanomaterials can provide adaptive responses to induce drug responses when combined with radiotherapy. Currently, several nanomedicine-based therapeutics have also been approved for preclinical studies and phase I/II/III clinical trials (Table 3). There has been relatively little exploration of nanotechnology in radiation oncology compared to chemotherapy or preoperative treatment. Additionally, it is crucial to elucidate their anticancer performance through the optimization of rational nanomaterial designs and their potential clinical translation is urgently required through future clinical studies.

## Figures and Tables

**Table 1:** Several studies delineated the significant implications of nanoparticles in the form of formulations in in vitro/in vivo models of cancers to enhance the efficacy of radiotherapy. ACF: acriflavine, Au: gold, Bi: bismuth, Lu: lutetium, Hf: hafnium, TPZ: tirapazamine.

Authors	Core Element	Nanoparticle Platform	Drug	Indication (Diseases)	Model	Reference	Conclusion
Zhuang M. et al.	Au	AuNPs-si-SP1		In vitro/in vivo	Lung cancer/A549 cell	[82]	AuNPs-si-SP1 increases the radiosensitivity of lung cancer both in vitro/in vivo by upregulating GZMB
Chiang C. et al.	Au	Hac-Au@SiO_2_		In vitro/in vivo	Glioblastoma multiforme	[85]	Hac-Au@SiO_2_ can effectively target CD44-expressing GBM cells to perform cancer cell targeted RT
Bhattarai S. et al.	Au	CXCR4 antibody conjugated Au nanoparticles (GNPs)		In vitro/in vivo	Human breast cancer lines/MCF-7, HTB-123, MDA-MB-231	[93]	CXCR4-targeted GNPs can enhance the efficacy of RT against TNBC by increasing oxidative stress and DNA damage
Zang Y. et al.	Bi	PVP−Bi_2_WO_6_		In vitro/in vivo	Hela cell/human umbilical vein endothelial cells	[100]	PVP−Bi_2_WO_6_ can effectively increase cellular DNA damage and colony formations under X-ray irradiation
Cheng X. et al.	Bi	BI_2_S_3_		In vitro/in vivo	4T1 cells	[101]	BI_2_S_3_ nanorods generate a strong synergistic effect by combining hyperthermia and nanoparticle-enhanced RT
Jeroen G. et al.	Lu	^177^Lu-labelled star polymers		In vivo	Subcutaneous CT26 isografts	[102]	^177^Lu-labelled star polymers can be used as potential probes for the passive delivery of radionuclides for endoradiotherapy
Zhong D. et al.	Au	α-Fe_2_O_3_@Au		In vitro	Murine breast cancer cells	[138]	α-Fe_2_O_3_@Au integrated with MRI, photothermal therapy and radiosensitization is a promising multifunctional theranostic nanomedicine for clinical applications
Liu Y. et al.	TPZ	Upconversion nanoparticles (UCNPs)	Tirapazamine (TPZ)	In vitro/in vivo	Hela cell/human cervical carcinoma cell	[141]	The UCNPs are highly hypoxia-specific, radiosensitive and cytotoxic and these substantially enhance the radiotherapeutic efficacy for targeting solid tumors
Xia D. et al.	Au	Au-Hb@PLT	Hemoglobin	In vitro/in vivo	Hela cell/human cervical carcinoma cell	[144]	Au-Hb@PLT can work as an oxygen vehicle and offers a promising approach to mitigate hypoxia and improve RT efficacy with a low RT dosage
Sang W. et al.	Hf	Hb@Hf-Ce6	PD-1 blockage	In vitro/in vivo	Melanoma cell, colon carcinoma cell, and mammary carcinoma cell	[146]	Hb@Hf-Ce6 nanoplatforms function as a new therapeutic option for cancer treatment through highly efficient X-ray-mediated RT-RDT in combination with immunotherapy
Chen Y. et al.	Hf	MnTCPP-Hf-FA MOF NPs		In vitro/in vivo	Melanoma tumor	[152]	MnTCPP-Hf-FA MOF NPs can effectively overcome hypoxia-induced radioresistance and prevent postoperative recurrence in vitro/in vivo experiments
Zhou X. et al.	ACF	Cu_2_-cSe@PtSe (CSP)		In vitro/in vivo	4T1 tumor cells	[163]	The synthesized Cu_2_-cSe@PtSe (CSP) can arrest the cell cycle of tumor cells to enhance their sensitivity to X-rays and decompose endogenous H_2_O_2_ into O_2_ to alleviate hypoxia and increase the generation of reactive oxygen species
Chen W.H. et al.	HIF-1α siRNA	Lipid-calcium-phosphate (LCP) nanoparticles		In vitro/in vivo	SCC4/SAS cells	[152]	The LCP can function as an efficient delivery of siRNA -HIF1α into tumors as part of a combination therapy along with PDT in the treatment of oral squamous cell carcinoma
Dong Z. et al.	Ferrous ions/BSO	BSO/GA-Fe(II)@liposome		In vitro/in vivo	4T1 tumor cells	[172]	The BSO/GA-Fe(II)@liposome works as an efficient adjuvant nanomedicine to promote clinically used conventional cancer chemotherapy and radiotherapy, by greatly amplifying the intratumoral oxidative stress
Li D. et al.	Folate	Folate-conjugated active targeting olaparib nanoparticles (ATO)		In vitro/in vivo	Hela cell/human cervical carcinoma cell	[173]	ATO represents a novel formulation for olaparib delivery and has promising potential for treating tumors with an overexpression of folate receptors

**Table 2:** Surface-modified nanoparticles with active targeting drugs/other radiosensitizing agents to enhance the combinatorial implications when combined with radiation therapy against cancer cells to maximize cancer cell ablation, ROS production, and to enhance the radiation dose efficacy.

Drug/Surface Modifier	Pharmacological Action	In Vitro/In Vivo Model	Conditions	Conclusion	References
CC225	Blocks epidermal growth factor receptor leading to impediment of VEGF production and cancer progression	-	Normal	Improvement in radiation-induced therapy response against tumors by C225 antiepidermal growth factor receptor antibody	[115]
Folate	Acts on folate receptor	Cervical cancer cell line (Hela) Glioma cell line (C6)	-	Folate-conjugated nanoparticles are one of the significant strategies which can be used as interventions in targeted cancer therapy	[219]
Celotoxib	Exerts its action by acting on COX-2 pathway	-	Normal	The ultra-small FA-AuNCs exhibited significant targeting efficacy for intracranial glioma tumors and an undeniable effect on increase of brain tumor-bearing rats’ survival time	[220]
Pentoxifylline	Blocks G2/M phase and delays DNA repair and mitosis; impedes G2/M block and induces delay in DNA repair and mitosis	-	Hypoxic	The synthetic complex has a potential role as an antioxidant agent in counteracting oxidative stress	[221]
Trastuzumab	Acts on HER2 receptor	Breast cancer cell lines (MDA-MB-361,BT-474, SK-BR-3)	-	Trastuzumab-AuNP-^177^Lu enables an efficient local radiation treatment of HER2-positive BC	[222]
Perifosine	Elevates radiation-induced programmed cell death	-	Normal	Perifosine enhances radiation-induced cytotoxicity in vitro/vivo experiments	[223]
Glucose	Exerts its action by acting on GLUT receptors	Human leukemia monocytic cell line (THP-1)	-	Glu-GNPs enhance the cancer killing of THP-1 cells 20% more than X-ray irradiation alone and GNP treatment alone	[224]
Gemcitabine	Acts by causing the arrest of S-phase	-	Normal	The synthesized PLGANPs loaded with gemcitabine and SPION can function as a radiosensitizer system which potentially could be used in RT	[225,226]
Co-grafted galactose (GAL)	Exerts its action by acting on asialoglycoprotein receptor	Liver carcinoma cell line (HepG2)	-	TheGAL-PEG-GNPs induce better radiosensitization through the apoptosis activated by free radicals induced by GNPs	[227]
Olaparib	Acts by slowing down DNA repair process	-	Normal	PARP inhibitors might be applicable to a wide therapeutic range of LET radiation	[228]
Cell penetrating peptides (CPPs)	Acts on plasma membrane and membrane-associated proteoglycans	Human colon adenocarcinoma cell line (LS180)	-	R8-modified GNPs efficiently enhance radiosensitivity of LS180 cells by arresting cell cycle and inducing apoptosis, with elevated ROS identified as the likely initiator	[229]
Nuclear localization sequences (NLS) peptide	Exerts its action by acting on nuclear pore complexes (NPC)	Cervical cancer cell line (Hela)	-	Peptide-modified gold NPs improved the efficacy of radiation therapy	[230]
*Salmonella enterica* serovarTyphi strainTy21a	Exerts its action by acting on tumor hypoxic regions	Colon carcinoma cell line (CT-26)	-	Vorinostat augm ents the anti-tumor effects of RT by abrogating radioresistance responses of PaCa cells	[231]
Vorinostat	Blocks on-homologous end joining (NHEJ) and homologous recombination (HR) NA repair system and repeals EGFR and NF-*κ*B signaling	-	Normal	AuNP-NUAP-STAT3d system induced an overall stronger radiosensitization effect in head and neck cancer cells	[232]
nucleolinaptamer (NUAP)	Exerts its action by acting on nucleolin	Epidermoid carcinoma (A431), Hypopharyngeal tumor of a squamous cell carcinoma (FaDu)	-	The ^177^Lu-T-AuNP is a significant radiation nanomedicine which can induce cell death of EGFR-positive TNBC	[233]
Panitumumab	Exerts its action by acting on Epidermal growth factor receptor (EGFR)	Breast cancer cell lines (MDA-MB-468, MDA-MB-231, MCF-7)	-	The gold NPs conjugated to pHLIP resulted in decrease in cell survival with radiation therapy	[38]
pH (low) insertion peptides (*pHLIPs*)	Acts at acidic pH of tumor microenvironment	Lung cancer cell line (A549)	-	Targeted delivery of a high gold-pHLIP in payload specifically to EGFR-(+)tumor cells which resulted in improved radiotoxicity for these tumor cells	[234]
*Epidermal growth factor* (*EGF*)	Exerts its action by acting on Epidermal growth factor receptor	Breast cancer cell lines (MDA-MB-468, MCF-7)	-	EGF-coated gold NPs led to a higher nanodelivery to promote the radiation therapeutic efficacy in EGFR-positive cancers	[235]
RGD (arginine, glycine, and aspartate polypeptide polymer)	Exerts its action by acting on Transmembrane heterodimeric αvβ3 integrin receptor	Breast cancer cell line (MDA-MB-231)	-	RGD/P-AuNPs induced the radiosensitization and targeted the integrin-overexpressing breast cancer cells and minimized their invasiveness	[236]

**Table 3:** Several studies describe the efficacy of high Z-metal elements in the form of nanoformulations for enhancing the efficacy of radiotherapy or brachytherapy in clinical trials. Fe: ferrous, Au: gold, Bi: bismuth, Lu: Lutetium, Hf: hafnium, Gd: gadolinium.

Authors	Core Element	Platform	Phase	Indication (Diseases)	Model	Reference	Conclusion
Bonvalot S. et al.	Hafnium	NBTXR3	Phase II/III	soft tissue sarcomas		[203,204]	The NBTXR3 activated by radiotherapy could represent a new treatment option in patients with locally advanced soft-tissue sarcoma.
Yadav M.P. et al.	Lu	^177^Lu-DKFZ-PSMA-617	Phase II/III	metastatic castration-resistant prostate cancer (mCRPC)		[208]	^177^Lu-DKFZ-PSMA-617 radionuclide therapy is a safe and effective approach in the treatment of mCRPC patients.
Violet J. et al.	Lu	^177^Lu-PSMA-617	Phase II	metastatic castration-resistant prostate cancer		[209]	^177^Lu-PSMA-617 enhanced therapeutic efficacy with minimal toxicity in men who underwent metastatic castration-resistant prostate cancer who have progressed after standard therapies.
Hofman M.S. et al.	Lu	^177^Lu-PSMA-617	Phase II	metastatic castration-resistant prostate cancer		[210]	^177^Lu-PSMA-617 resulted a potential adjuvant life-prolonging treatment option for men with mCRPC.
Tagawa S.T. et al.	Lu	^177^Lu-J591	Phase II	metastatic castration-resistant prostate cancer		[211]	A single dose of ^177^Lu-J591 was well tolerated with reversible myelosuppression.
Maier-Hauff K.	Fe	magnetic iron-oxide	Phase II	recurrent glioblastoma multiforme		[212]	Thermotherapy using magnetic NPs in conjunction with a reduced radiation dose is safe and effective and leads to longer OS-2 compared to conventional therapies in the treatment of recurrent glioblastoma.
Verry C. et al.	Gd	AGuIX	Phase I	brain metastases		[213]	Combining AGuiX with radiotherapy for patients with brain metastases is safe and feasible.
